# Integrating Genomics and Clinical Data for Statistical Analysis by Using GEnome MINIng (GEMINI) and Fast Healthcare Interoperability Resources (FHIR): System Design and Implementation

**DOI:** 10.2196/19879

**Published:** 2020-10-07

**Authors:** Julian Gruendner, Nicolas Wolf, Lars Tögel, Florian Haller, Hans-Ulrich Prokosch, Jan Christoph

**Affiliations:** 1 Department of Medical Informatics Friedrich-Alexander University, Erlangen-Nürnberg Erlangen-Tennenlohe Germany; 2 Diagnostic Molecular Pathology Institute of Pathology Friedrich-Alexander University, Erlangen-Nürnberg Erlangen Germany

**Keywords:** next-generation sequencing, data analysis, genetic databases, GEnome MINIng, Fast Healthcare Interoperability Resources, data standardization

## Abstract

**Background:**

The introduction of next-generation sequencing (NGS) into molecular cancer diagnostics has led to an increase in the data available for the identification and evaluation of driver mutations and for defining personalized cancer treatment regimens. The meaningful combination of omics data, ie, pathogenic gene variants and alterations with other patient data, to understand the full picture of malignancy has been challenging.

**Objective:**

This study describes the implementation of a system capable of processing, analyzing, and subsequently combining NGS data with other clinical patient data for analysis within and across institutions.

**Methods:**

On the basis of the already existing NGS analysis workflows for the identification of malignant gene variants at the Institute of Pathology of the University Hospital Erlangen, we defined basic requirements on an NGS processing and analysis pipeline and implemented a pipeline based on the GEMINI (GEnome MINIng) open source genetic variation database. For the purpose of validation, this pipeline was applied to data from the 1000 Genomes Project and subsequently to NGS data derived from 206 patients of a local hospital. We further integrated the pipeline into existing structures of data integration centers at the University Hospital Erlangen and combined NGS data with local nongenomic patient-derived data available in Fast Healthcare Interoperability Resources format.

**Results:**

Using data from the 1000 Genomes Project and from the patient cohort as input, the implemented system produced the same results as already established methodologies. Further, it satisfied all our identified requirements and was successfully integrated into the existing infrastructure. Finally, we showed in an exemplary analysis how the data could be quickly loaded into and analyzed in KETOS, a web-based analysis platform for statistical analysis and clinical decision support.

**Conclusions:**

This study demonstrates that the GEMINI open source database can be augmented to create an NGS analysis pipeline. The pipeline generates high-quality results consistent with the already established workflows for gene variant annotation and pathological evaluation. We further demonstrate how NGS-derived genomic and other clinical data can be combined for further statistical analysis, thereby providing for data integration using standardized vocabularies and methods. Finally, we demonstrate the feasibility of the pipeline integration into hospital workflows by providing an exemplary integration into the data integration center infrastructure, which is currently being established across Germany.

## Introduction

### Background

Combining omics data with other clinical patient data has been the focus of multiple studies in the past years [1-3]. In particular, the emergence and widespread use of next-generation sequencing (NGS) for the identification of pathological gene variants has led to an increase in the amount of data available for diagnosis. This has directly improved the quality of medical care for many diseases [4-6]. The meaningful combination of NGS data with other patient data from varying sources has been challenging for a number of reasons. First, the amount of data generated by NGS is vast compared to other data such as laboratory and clinical data, data derived from classical diagnostic tests, and demographic data, for example, age and gender. Second, NGS data typically contains many data points, which might not be relevant for subsequent analysis. Third, NGS data are highly sensitive in terms of privacy and more difficult to anonymize in a meaningful way. Therefore, any analysis within and across institutions will have to be carefully considered and crafted in a privacy-preserving manner. Many different studies have been conducted to investigate the feasibility of directly integrating omics data into clinical data repositories such as OMOP (Observational Medical Outcomes Partnership) [7], i2b2 (informatics for integrating biology and the bedside), and transMART [2]. The OHDSI-OMOP (Observational Health Data Sciences and Informatics-OMOP) common data model (CDM) focuses on observational research, and i2b2 focuses on the integration of different types of data into 1 clinical repository. These systems require an extra genomics pipeline to be run before the data can be loaded and integrated into the data repositories. Many of the proposed methods are not optimized for initial data investigation, automatic annotation of the data, or privacy-preserving cross-hospital analysis.

This study focusses on bridging this gap by creating a system that supports the whole process. It starts from variant call format (VCF) files. The data are then annotated and preanalyzed. In the final step, selected genomics data are combined with other structured and standardized patient data in 1 data set in a table format for further statistical analysis. We achieve this by extending the open source framework GEMINI (GEnome MINIng) [8]. It allows a user to load VCF files and places genetic variants, sample phenotypes and genotypes, as well as genome annotations into 1 database. This supports powerful exploration of genetic variations for disease and population genetics. GEMINI makes it possible to analyze the NGS data using structured query language (SQL), which allows researchers to filter variants by clinical relevance, rarity, and read quality. Our proposed system then combines GEMINI with the Fast Healthcare Interoperability Resources (FHIR) [9], a standard for health care data exchange. FHIR describes the clinical information in so-called resources. These modular components describe different elements found in electronic health records, for example, a patient or a clinical observation such as a laboratory result. It was developed to address the shortcomings of the previously developed HL7 clinical care document standard. The FHIR standard aims to improve interoperability, and its lightweight nature and direct use of common data formats such as JSON and XML allows it to easily integrate with lightweight web services, similar to the ones created in the pipeline described here.

In order to create a pipeline on standardized data such as FHIR, heterogenous hospital data need to be transformed into a standardized format first. The German Medical Informatics Initiative (MI-I) [10] has recently funded 4 consortia of mainly university hospitals across Germany to investigate how heterogenous clinical data, including omics data, can be integrated into clinical data repositories. One aim of the MI-I is to establish data integration centers (DICs), which are the backbone of the cross-hospital and cross-consortia communication. The DICs will provide different services, including data integration, data harmonization, standardized data repositories, consent management, and ID management [11-14]. This study builds on efforts of 2 use cases from the MIRACUM (Medical Informatics in Research and Care in University Medicine) [14], particularly on 1 use case, which aims to establish a genomics pipeline to support NGS data interpretation and clinical decision making at molecular tumor boards. This MIRACUM-Pipe [15], similar to other genomics pipelines, creates VCF files in the process. These VCF files provide a good point of integration for further data analysis beyond the use for the molecular tumor board. Another use case of the MIRACUM consortium focuses on analyzing data within and across hospitals in a privacy-preserving manner, as well as deploying prediction and decision support models in a clinical context.

### Aim of the Study

In order to treat individual patients within the molecular tumor board, individual sequencing data are generated in VCF. Our study supports research by building on these data, aggregating them across multiple patients and enriching them with clinical data. These integrated data sets are analyzed with open-source analysis tools and frameworks to generate or validate hypotheses. Our proposed system extends GEMINI with multiple web services and a user interface to support the whole data flow as well as the subsequent integration with the already established FHIR [9] data repository, KETOS [16], and DataSHIELD [17] platforms for statistical analysis. To validate our approach, we implemented and applied this data analysis pipeline to data from the 1000 Genomes Project, which yielded highly consistent results. We then applied our pipeline to high-throughput gene panel sequencing data from a cohort of 206 patients from the Institute of Pathology of the University Hospital Erlangen and compared the findings with the results of the established Illumina genomics pipeline [18]. Finally, as a proof of concept, we combined the selected data from this patient cohort with diagnosis codes from the local FHIR server and analyzed the correlation between locations based on the diagnoses and gene mutations to demonstrate one potential use of the system.

## Methods

### Common Requirements for NGS Analysis

To understand the requirements for NGS analysis, we studied the status quo of the Institute of Pathology of the University Hospital Erlangen. The institute analyses gene mutations by using a commercial pipeline provided by Illumina and presents its findings to the local molecular tumor board. A detailed description of the DNA and RNA library preparations from patient tissues can be found in Multimedia Appendix 1. The pipeline currently used by the institute has limited capabilities when it comes to performing cross-patient analysis and is not easily integrated with other open-source solutions. Therefore, the following requirements were defined, which need to be met: be on-premises, be open-source, deliver the same results as a commercial product, allow analysis across multiple patients, and possibility of web-based integration with existing and new infrastructure to be developed.

### Data Analysis

**Figure 1:**
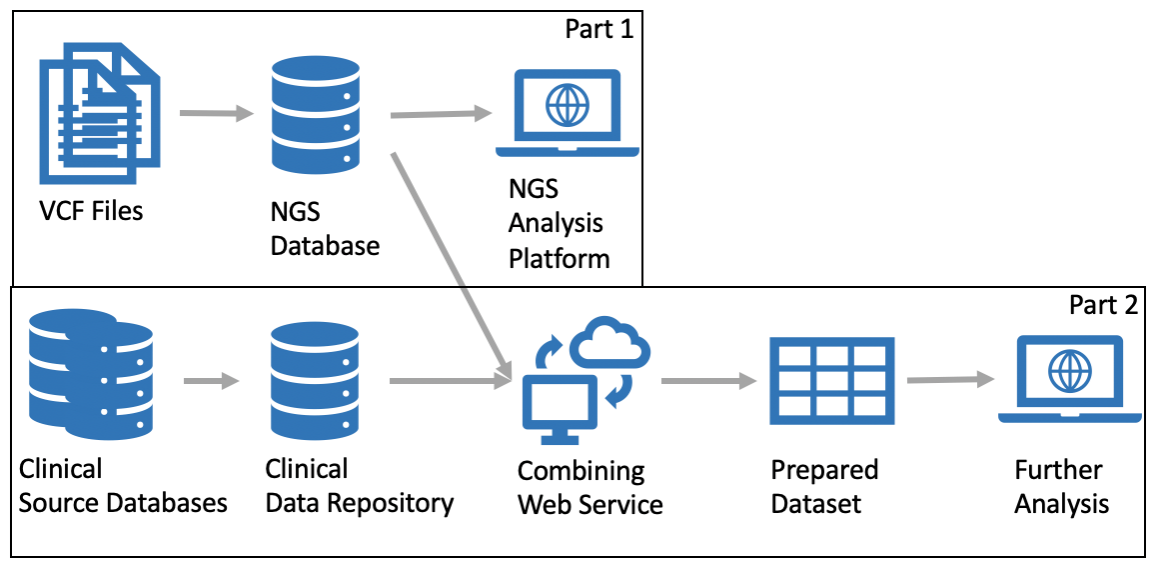
Genomics pipeline and analysis system. NGS: next-generation sequencing; VCF: variant call format.

The biggest drawback of the current system is that it does not support cross-patient analysis and integration of additional patient data, as the patient data are distributed across multiple systems in the hospital. The system therefore does not currently support a way to, for example, correlate a patient’s diagnosis with particular gene mutations as is done in our exemplary analysis shown below. We therefore propose a new pipeline, which improves on these drawbacks. Figure 1 depicts the multistep analysis workflow of such a genomics pipeline. In the first step, VCF files are uploaded into a database for gene annotation and, subsequently, gene variant filtering and analysis. To allow further processing of the data, it is crucial to filter variants that are irrelevant for a particular research question. The amount of data and noise must be reduced. For this, researchers need to be able to query and filter the genomic database. This would satisfy any research only focusing on NGS data; however, in a clinical setting, NGS data become truly meaningful when clinical patient data are available. To achieve this, a standardized way to combine genomics data with clinical data is essential. Finally, combined data needs to be transformed to a common format that can easily be analyzed, processed further, and integrated into existing data analysis frameworks. The resulting system described in Figure 1 can be split into 2 larger parts: (1) Part 1: an NGS processing and analysis pipeline and (2) Part 2: a system for integrating clinical patient data for further analysis.

#### Part 1: NGS Processing and Analysis Pipeline

**Figure 2:**
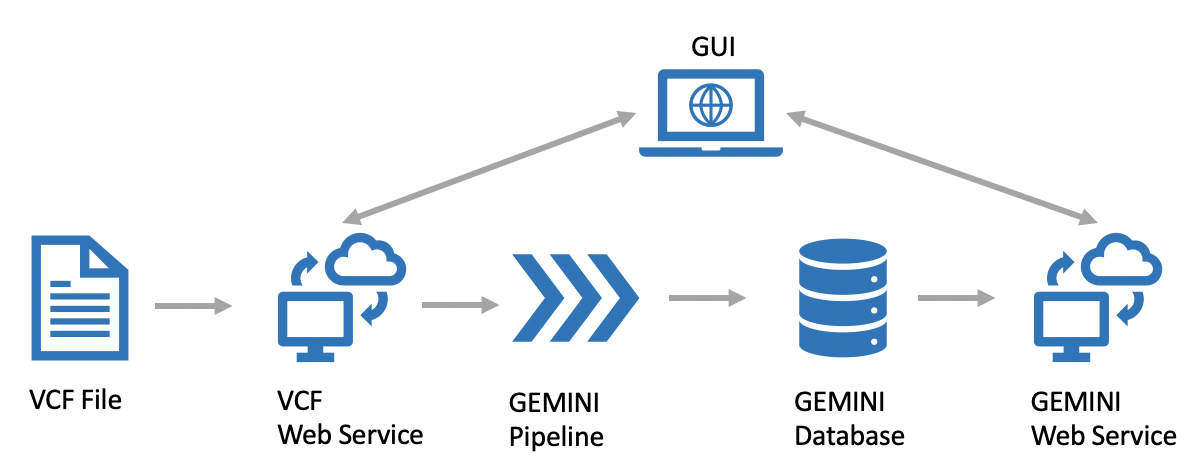
Part 1: Next-generation sequencing processing and analysis pipeline architecture. GUI: graphical user interface; VCF: variant call format; GEMINI: GEnome MINIng.

**Figure 3:**
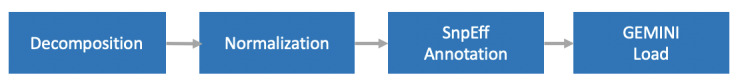
GEMINI pipeline schema. SnpEff: single nucleotide polymorphism effect; GEMINI: GEnome MINIng.

Figure 2 shows the first part of the architecture of the implemented genomics pipeline and analysis system. The proposed system includes a graphical user interface (GUI, Figure 2), which supports all steps. The GUI is used to upload a VCF file via the VCF web service, which is then processed and loaded into GEMINI via the GEMINI web service. The web service then uses our integrated GEMINI pipeline (Figure 3) to process and load the data into GEMINI.

This GEMINI pipeline (Figure 3) first decomposes the VCF file using the Variant Tools software [19] decompose functionality, which splits up multiallelic variants of a VCF file, resulting in a separate row for each reference sequence/alternative sequence pair. The decomposed VCF file is then normalized using the normalize function of the Variant Tools software. This step ensures that each VCF entry is left aligned and parsimonious, as described by Tan et al [20]. The resulting VCF file is then automatically annotated using the SNP (single nucleotide polymorphism) effect (SnpEff) tool, reported by Cingolani et al [21]. The SnpEff annotation process enriches the VCF file with information about possible effects or malignancies. One important functionality of GEMINI is the addition of multiple extra annotations during the load process. This is the last step of our GEMINI pipeline (GEMINI-Load, Figure 3) and includes the following annotation sources: 1000 Genomes Project (population data, allele frequencies) [22], dbSNP (reference snp IDs according to the National Center of Biotechnology Information) [23], and ClinVar (disease information, eg, disease name or clinical relevance) [24].

**Figure 4:**
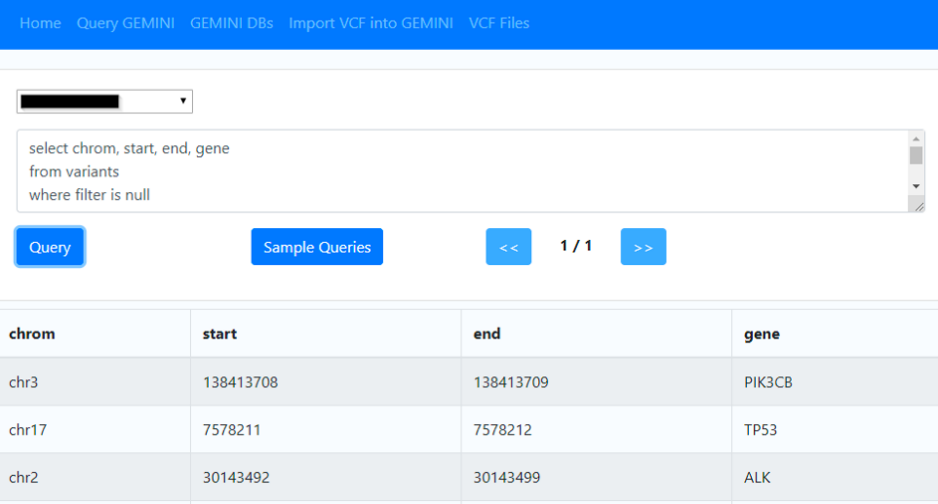
Exemplified user interface.

Once loaded, data can be analyzed using the user interface (GUI, Figure 4) and the GEMINI web service. The user interface allows one to download a GEMINI database in the form of an SQLite file for further use. It further implements a web-based query tool for the GEMINI database. The advantage of this web-based query tool is that the researcher does not need to learn how to use the command line tool, while still providing full GEMINI query functionality.

#### Part 2: A System for Integrating Clinical Patient Data for Further Analysis

**Figure 5:**
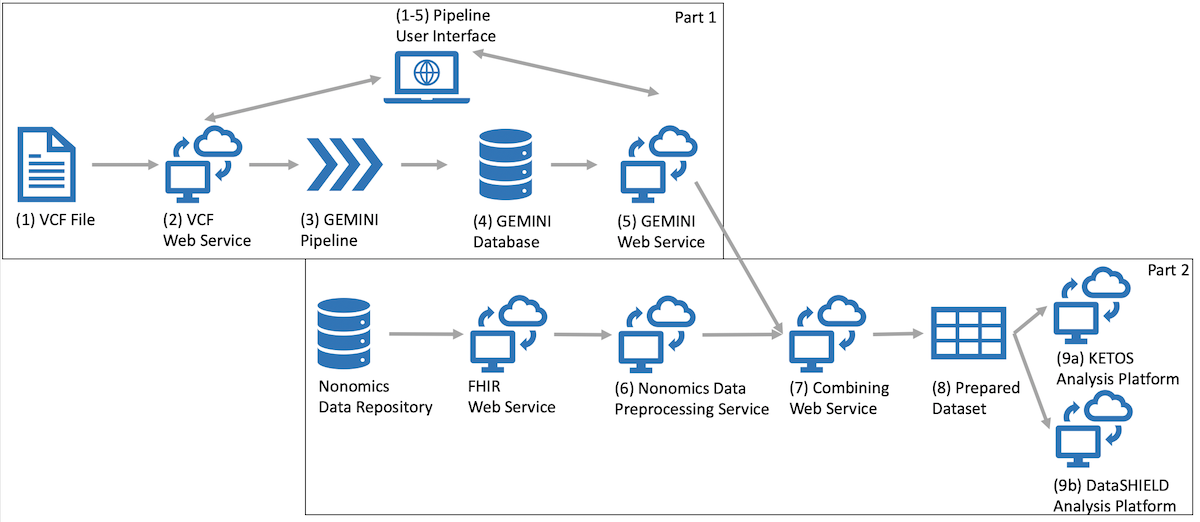
Combining genomics and patient data. VCF: variant call format; GEMINI: GEnome MINIng; FHIR: Fast Healthcare Interoperability Resources.

Figure 5 depicts the structure of the complete system (numbers indicate the order of the executable steps). A VCF file (Step 1) is read, annotated, and loaded into GEMINI (steps 2-4). Then, NGS data are combined with nonomics, clinical patient data in an FHIR format using the patient ID as a link in a prepared data set (steps 5-8) for the final analysis. The combining of the NGS and clinical patient data is performed by the combining web service, which is described in more detail with an example in the Results section. One of the advantages of an on-premises open source solution is the straightforward extendibility with the other already established web services. Here, we used the KETOS analysis platform established as part of the local DIC. Further, the creation of a prepared data set in the form of a comma-separated values (CSV) file allows a direct import into the DataSHIELD platform for privacy-preserving cross-hospital analysis (steps 8, 9a, and 9b). DataSHIELD supports this as its underlying data warehouse supports the loading of CSV files for further analysis.

## Results

### Overview

We implemented and deployed the above-described architecture at the University Hospital Erlangen. Its functionality was then validated by comparing the results of our analysis system to the results from the 1000 Genomes Project [22] and subsequently by assessing the ability of the system to identify relevant tumor-associated gene variants. Finally, we used the system to combine gene mutation data from 206 patients with diagnosis codes from the local FHIR data repository. We analyzed the resulting prepared data set by using KETOS and Jupyter Notebook (interactive cell-based code development in a web browser) [25].

### Comparison with the 1000 Genomes Project

To demonstrate the accurate functionality of the pipeline, we investigated the genomic variations of the X-chromosome in a cohort of 1092 individuals, which were studied in the initial phase of the 1000 Genomes Project [26]. First, we loaded the publicly available VCF file data supplied by the study into a GEMINI database by using our pipeline. Then, we compared the variation data in our database to the results from the 1000 Genomes Project [27] (Table 1).

**Table 1:** Comparison of the statistical evaluation of the 1000 Genomes Project and GEMINI (GEnome MINIng) pipeline.

Mutations			1000 Genomes Project (n)		GEMINI (n)
**Single nucleotide polymorphisms**					
	Total variants	~1,300,000		1,275,275	
	Average per sample	~105,000		104,757	
**Indels**					
	Total variants	~59,000		59,157	
	Average per sample	~13,000		12,715	
**Large deletions**					
	Total variants	432		432	
	Average per sample	26		26	

As shown in Table 1, results generated with our system were identical to the results generated by the 1000 Genomes Project. The analysis, including converting and loading the data into GEMINI, took approximately 3 hours to run on a MacBook Pro (15-inch, 2016)-System with 2.7 GHz Intel Core i7-Processor and 16 GB 2133 MHz LPDDR3-RAM. Once loaded, the SQL queries took approximately 120 seconds to complete. This clearly demonstrates that the bottleneck is loading the data rather than the subsequent analysis using GEMINI.

### Comparison With Illumina Pipeline and Genomic Analysis

#### Single Patient Analysis

We loaded individual patient–derived VCF files into the GEMINI database and prepared an SQL query according to established filter criteria used in the Illumina pipeline at the Institute of Pathology. In the first iteration, the resulting SQL query (Multimedia Appendix 2) filtered the following: all variants that failed to pass any of the variant quality filters, all variants that displayed a low impact in regard to protein functionality, and all variants that were not considered pathogenic as they did not change the coding protein (eg, synonymous variants, intronic variants, upstream/downstream variants). In addition, all variants with a population-based allele frequency of ≥2% were also excluded. Table 2 shows an exemplary comparison of the results from the Illumina versus the GEMINI pipeline. Illumina VariantStudio yielded 16 variants, while GEMINI yielded 15 variants. The Illumina VariantStudio included chromosome 4 position 10085736. GEMINI also included chromosome 9 position 21970916.

**Table 2:** Comparison of the filtered results of the mutations in the GEMINI (GEnome MINIng) and Illumina pipeline.

Chromosome	Position	Codon change (according to the Human Genome Variation Society coding)	Illumina	GEMINI
1	40366658	c.539A>G	✓	✓
1	40366659	c.537_538insCG	✓	✓
2	215632255	c.1518_1519delTGinsCA	✓	✓
3	12645693	c.776C>G	✓	✓
4	106156187	c.1088C>T	✓	
5	112178795	c.7504G>A	✓	✓
5	176520270	c.1189_1190delGGinsAC	✓	✓
5	176522605	c.1702_1704delCCAinsGCC	✓	✓
7	116411990	c.3029C>T	✓	✓
9	21970916	c.442G>A		✓
16	3778424	c.6624A>C	✓	✓
16	3779338	c.5709dupG	✓	✓
16	3779338	c.5709delG	✓	✓
16	3779361	c.5687A>C	✓	✓
17	7579408	c.277_278delCT	✓	✓
22	41546158	c.2773C>A	✓	✓

Based on this result, we revised the filter criteria (Multimedia Appendix 3) so that only variants with the filter impact_severity *high* or one of the impact types (disruptive_inframe_deletion, disruptive_inframe_insertion, missense_variant) were included in the results. Furthermore, the ClinVar database was used to assess the pathogenicity of individual gene alterations, which is readily available in the annotated GEMINI database. All mutations that were classified as benign, likely_benign, and benign/likely_benign according to the ClinVar database were deleted from the data set. Mutations without known ClinVar status were kept and manually classified by interrogating web-based variant repositories. The adjusted SQL query yielded the same results as the Illumina pipeline, which qualifies the open source solution for routine use in clinical practice. Finally, the automation of the filtering steps saves hands-on time of approximately 30 minutes per patient analysis.

#### Multiple Patient Analysis

We used gene sequencing data of 206 patients from the University Hospital Erlangen. To analyze the data, the 206 individual VCF files were first merged and then loaded into GEMINI. Each file had a size of 57 MB, which adds up to a total file size of 12 GB. The merged VCF file had a size of 4.8 GB, and the GEMINI database a size of 664 MB. To analyze the whole patient cohort, we first created an SQL query to provide simple descriptive statistics of the cohort, such as the overall number of mutations by *impact_severity* and *type* (snp, indel). As expected, most mutations were classified with a low impact severity. We then further analyzed the merged data with another SQL query to determine the occurrence of gene mutations, impact, and codon_change (Multimedia Appendix 4). This analysis revealed that impact_severity is not a suitable filter for dichotomizing high impact variants from synonymous variants. Therefore, specific impacts (impact column) were used instead. In addition, the same filter criteria as described for the adjusted query were used for further analysis (Multimedia Appendix 3). Looking at the results in more detail, it became clear that many of the mutations were sequencing artefacts, which would have to be excluded before further analysis. Especially critical is the fact that merging of multiple VCF files leads to information loss and reduces the quality of the sequencing data. We discovered that the merge function vcf-merge of VCFtools only keeps the lowest passed quality filter for each variant. This means that a higher quality grade will only be listed in the FILTER column if it was passed by each sample for a variant. Other merge functions such as the merge function of BCFtools allow the user to choose between either keeping the lowest passed or the highest passed filter for each variant. However, in all cases, the quality criteria of each individual sample cannot be reconstructed. This poses a potential problem for further analysis as 1 low-quality read for a variant in 1 sample would obscure potential good results in others, leading to the exclusion of good-quality reads; vice versa, 1 high-quality read may mask low-quality reads, which might impact the data analysis. A solution for this problem is to either exclude particularly poor-quality samples before joint analysis is executed or to load and analyze samples individually in an automated way. This will preserve sample quality information in a combined data set.

### Creating a Prepared Data Set for Combined Analysis

To create a combined prepared data set and to make it available for further analysis, we integrated the combining web service with the KETOS platform by making the web service, which combines the NGS data with the nonomics data, available to the KETOS host by using internet protocol address restriction as well as password protection. The combined prepared data set is a combined subset of all the available NGS and clinical data, which were prepared for 1 specific research question. The combining web service data for a particular research question is identified using JSON syntax. It includes an *fhir* part to specify patient data in the form of the required FHIR resources and a GEMINI part to specify the filtered variants to be included. The specification also requires patient IDs to identify the patient cohort for further analysis (see Multimedia Appendix 5). In an initial combining web service specification, we required the user to specify the exact variant positions and alleles. This returned a data set in which the variant-specific fields are connected to the variant via a prefix, which is added to all extracted variant fields. The prefix contains the chromosome, chromosomal location of the variant, as well as the nonreference allele, for example, chrX#154158284#154158285#C#. The web service collects the data for the prepared data set and returns the data either as a JSON array or as a CSV file. The resulting data set in the CSV format is then created as shown in Table 3. It contains 1 row for each patient. Each row includes the patient ID as well as other fields specified by the user.

**Table 3:** Example of a prepared data set.^a^

Patient_ID	Gender	Date of birth	Disease	<prefix>^b^ ref	<prefix>^b^ alt	<prefix>^b^ gts
28	male	01.10.41	1	G	C	C

^a^This table shows only the examples of values.

^b^<prefix> is the concatenation of the chromosome, chromosomal location of the variant, and nonreference allele, eg, chrX#154158284#154158285#C#—this would, for example, result in the following column name: chrX#154158284#154158285#C#ref.

In our further analysis, we found this specification of the combining web service to be limiting. We therefore extended the web service to allow the researcher to specify the GEMINI query used to collect the NGS data directly in SQL, rather than being limited to extracting fields by variant position only. The resulting request specification is analogous to the one described in Multimedia Appendix 5, except for the following extending fields inside the GEMINI part: sql_mode, sql, columns, rows. This allowed us to create an example of a data set by using a query similar to that shown in Multimedia Appendix 6, which resulted in a data set similar to Table 4. This allowed us to avoid information loss regarding read quality, as the combining web service can query an individual database for each patient, thereby omitting data-merging steps. The resulting data set could then be imported into the KETOS analysis environment where further analysis could be conducted within a Jupyter Notebook [25] by using a researcher’s favorite tools. Further, the comma-separated nature of the data set means that it could also be imported into DataSHIELD, which supports further privacy-preserving analysis across institutions.

**Table 4:** Format of the initial raw data set.^a^

Patient_ID^b^	Gender^c^	Date of birth^d^	Diagnosis^e^ (ICD^f^10 Code)	Gene^h^
PSEUDO-ID-1	Female	01.01.50	2019-01-01T00:00:00+00:00	*PIK3CB*
PSEUDO-ID-2	Male	01.01.50	2019-01-01T00:00:00+00:00	*PIK3CB*
PSEUDO-ID-3	Male	01.01.50	-	-
PSEUDO-ID-4	Male	01.01.50	2019-01-01T00:00:00+00:00	-

^a^Since the combined data set comprised 206 patients, 135 diagnoses, and 152 genes, values shown in this table are only examples, as the entire data cannot be represented here.

^b^Examples of IDs of patients.

^c^Examples of genders.

^d^Examples of birth dates of patients.

^e^Example value, C20 or C61; timestamp diagnosis. (One column per diagnosis - if there is no diagnosis for a patient, the column will be empty in this patient's row.)

^f^International Classification of Diseases.

^h^Gene name examples. (One column per Gene - if there is no gene mutation for a patient, the column will be empty in this patient's row.)

### Exemplary Analysis of Tumor Entity and Diagnosis Inside KETOS by Using Jupyter Notebook

As a proof of concept for our system, we created a data set with combined diagnostics and tumor mutational data for further analysis. The initial data set included 206 patients. In the first step, we identified 135 cancer-related diagnosis codes across our cohort. We then used the revised GEMINI query (Multimedia Appendix 3) to collect the gene mutations across our cohort. The combined data set comprised 206 patients, 135 diagnoses, and 152 genes. The final raw data set had the format shown in Table 4.

**Figure 6:**
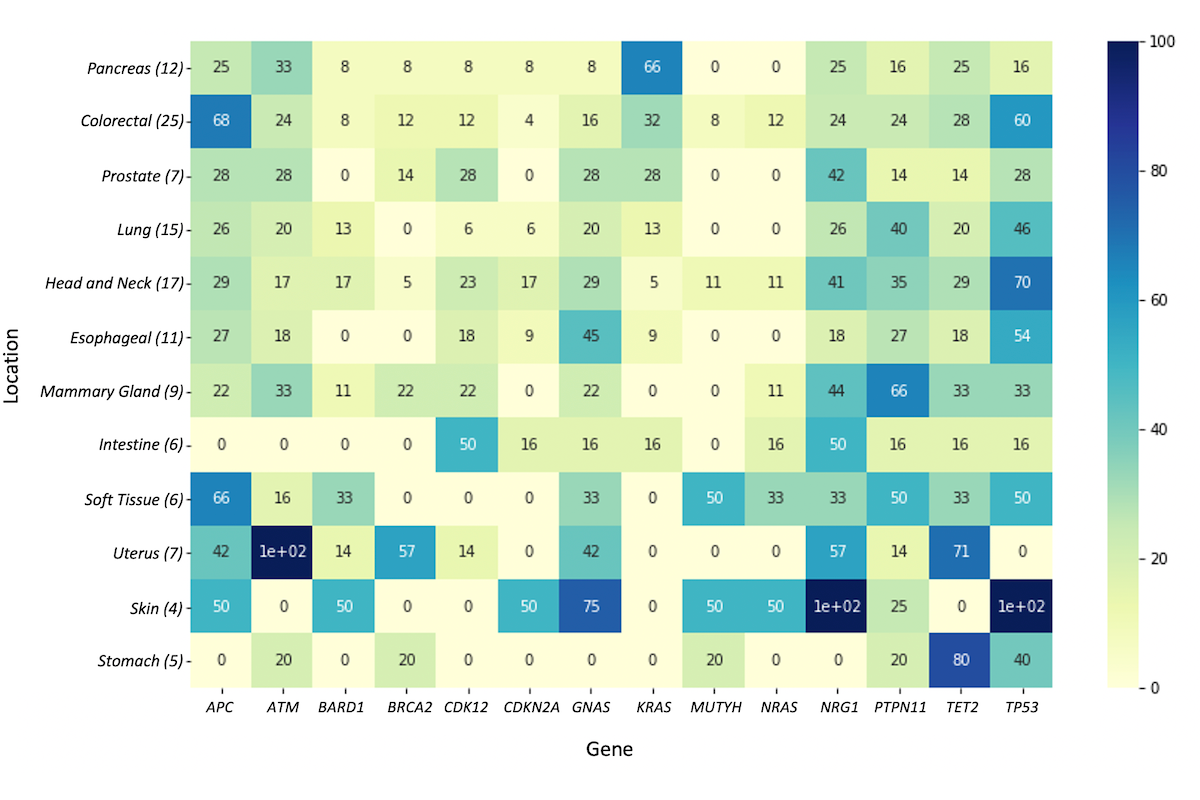
Distribution (%) of the gene mutations by location. Y-axis: location (number of patients); x-axis: gene, eg, 80% for stomach for *TET2* means that 4 of 5 patients with stomach cancer had a mutation in *TET2*.

Using Jupyter Notebook in one of the analysis environments inside the locally installed KETOS data analysis platform; we requested the data from our combining web service. Patients without diagnosis were removed. We removed all secondary tumor diagnoses from our data set and focused on primary tumor locations. Tumor entities represented by less than 4 patients were regarded as underrepresented and were also removed prior to further analysis. This yielded a sample group of 124 patients across 12 tumor entities. A total of 142 unique genes with mutations were identified across this population. As a proof of principle, we aimed to identify the driver mutations for each tumor entity. Therefore, we included only genes that were mutated in more than 50% of the patients, resulting in the heat map depicted in Figure 6. This filtering step identified 14 frequently mutated genes within the patient cohort, mainly *bona fide* tumor suppressor genes such as *APC*, *ATM*, *BRCA2*, or *TP53* and well-known protooncogenes such as *GNAS*, *KRAS*, and *NRAS*. Notably, this analysis revealed tumor characteristic mutational profiles. For instance, the colorectal cancer cohort displayed a characteristic rate of *APC*, *TP53*, *KRAS*, and *NRAS* mutations when compared to previously reported data [28]. Likewise, *KRAS* mutations were highly prevalent in the pancreatic cancer cohort, a malignancy with an observed *KRAS* mutation rate of up to 95% [29]. Since patients with cancers were distributed across multiple sites and the number of patients for some locations was small, a very accurate distribution by location might have been lost in the analysis process. This could be remedied using a larger sample size and factoring the multisite cancers into the analysis.

## Discussion

### Overview

We presented an open-source web-based NGS annotation pipeline based on the GEMINI database, which produced the same results as the established Illumina pipeline used in routine diagnostics. We then extended our pipeline to combine the annotated NGS data with other clinical data and demonstrated the feasibility of our approach in an exemplary analysis, which combined gene mutation data with clinical diagnosis codes.

The new pipeline improved on the current pipeline by supporting this cross-patient analysis. This allowed us to combine the NGS data with other patient data from other systems across the hospital in 1 data set. The source code of this project is open to the public and is available on GitHub [30].

Previous studies have investigated the integration of omics with other clinical data. In particular, 2 larger systems, which have recently seen efforts to achieve this integration, are the OHDSI-OMOP CDM and i2b2. Murphy et al [2] compared 3 different strategies for integrating omics data into i2b2, 2 of which combined omics and clinical data in the same database. The third approach is similar to the one described in this study. It kept omics data in a separate database. For the OMOP CDM, NGS data were integrated into 1 database with clinical data by creating additional tables for the omics data. All approaches had 1 thing in common that they did not provide a user interface for loading data into the respective databases. In contrast, our pipeline significantly improves usability by providing a user interface, which allows a user to process VCF files and load them into the GEMINI analysis database. Additionally, using an established omics database allowed us to fast-track querying and preanalysis of the genomics data, thereby significantly reducing the amount of data at an early stage in the process. GEMINI is part of the pipeline used by the MIRACUM consortium and therefore provides us with a clear point of integration. This means that once the MIRACUM-Pipe has been established across the different DICs, it can potentially replace the Illumina pipeline and integrate directly with the solution described here.

### Generalizability of the Pipeline

In this paper, we focus on the integration with the DICs currently being established across Germany. However, as the system relies on common formats (VCF for NGS data and FHIR for clinical patient data), it could easily be applied to other hospitals. The FHIR format, in particular, is currently seeing a rise in popularity and as electronic health record vendors will provide more and more data in the FHIR format, establishing a local FHIR store with patient data should be feasible for many institutions. However, as with any standardized format, studies that have captured data in their own format or another standard format such as OMOP would have to convert their data into FHIR formatted data in order to take advantage of this solution. As the whole solution can be deployed on premises, acceptance for the solution should be high as the deployment can be tailored to the specific needs of individual institutions and no data has to leave the institution at any time. In our example, we show how the prepared data sets, which result from the pipeline, can be analyzed in Jupyter Notebook and KETOS, a web-based analysis platform. Generally, the prepared data sets in the CSV format could be read into many other analysis platforms such as DataSHIELD, converted into table-like formats, and analyzed further.

### Potential Use of the System

We have demonstrated in this paper how our system can be used to combine diagnoses with NGS data for further analysis. Connecting the system to an FHIR repository of standardized data means that there is a great opportunity to undertake further analyses in the future with other data available in an FHIR store. The MIRACUM FHIR store at the University Hospital in Erlangen currently has information about patients, encounters, diagnoses, laboratory results, procedures, and medications. The system described here would, for example, support further investigation of the correlation between the diagnosis of COVID-19, disease outcome from hospital discharge information, and gene mutations. The prerequisite for this would be that the gene sequences of respective patients are sequenced as part of a wider investigation.

### Lessons Learned

We were able to augment the existing GEMINI software with web services. The resulting pipeline, which is based on the open source project, could be hosted on premises. It fulfilled the requirement of producing the same results as the established commercial pipeline. One of the biggest drawbacks of the GEMINI database is that it does not allow the uploading of the data of multiple patients directly into 1 database. Instead, data from multiple patients have to be merged first, which leads to a loss of information regarding the sequencing quality for a particular variant. This may impact analysis and data interpretation as low-quality reads and high-quality reads would be interpreted in the same way. However, this limitation can be augmented by preselecting files with good-quality reads or loading each file individually and focusing only on variants that are relevant to the particular research question. The combining web service in sql_mode described above can also be used for this type of analysis by simply requesting GEMINI data from individual databases. Once loaded into the GEMINI database, queries for a particular variant were very fast and results were returned within seconds, making the analysis across multiple patients feasible.

### Limitations

The web-based user interface provides direct access to different databases. However, exporting prepared data sets is currently not supported and has to be triggered using a web service representational state transfer call. Therefore, the platform presented here requires the user to be proficient with SQL and basic programing skills in order to extract and analyze the data, making it unsuitable for users with no programming or SQL skills. The chosen method of prefiltering data for further analysis using an established, fast open source tool allows one to avoid large data volumes. However, it also limits the explorative analysis of NGS with other patient data. The medical device regulations in Germany approves the use of the system described above for research purposes only. The MIRACUM DICs follow these regulations. The reliance on FHIR requires the infrastructure to provide an FHIR server. However, the MI-I initiative has already set FHIR as the format of choice for interconsortia communication [31].

### Future Directions

In this study, we focused on 1 hospital to show how a potential analysis can be made possible within a hospital. In the future, it would be of interest to duplicate the platform across multiple hospitals to establish cross-hospital analysis pipelines and run analyses across institutions by using DataSHIELD. Pipeline automatization, ie, automated variant annotation and execution of predefined variant filtering/classification steps as well as automated inclusion of results in clinical reports could lead to significant time savings. Another prospective extension would be the integration of therapy suggestions from different web-based databases such as Somatic Mutations In Cancer [32], My Cancer Genome [33], and ClinicalTrials.gov [34]. The prepared data set or combining web service should be integrated further into existing workflows to automate data selection and preparation as well as to make the data provision easier. The FHIR standard for patient data is constantly being developed and has been standardized to integrate omics data directly. Our approach could be extended to load selected data directly into an FHIR database, which would make explorative analysis easier.

### Conclusion

In this study, we successfully demonstrated how NGS genomics data can be combined with FHIR clinical data to provide accessory analysis to existing gene variant analysis solutions in clinical settings. The chosen method of prefiltering data for further analysis by using an established database, which is based on the fast GEMINI open source tool, means that large data volumes can be avoided. In addition, we showed that a pipeline and web services built on open-source tools delivered the same results as a commercial product and could be hosted on the premises, and be integrated well within a clinical DIC, building on existing structures, and benefiting from the data standardization DICs provide. Finally, we showed how the system could be used to create and analyze a data set, which included gene mutation and diagnosis data.

## Appendix

Multimedia Appendix 1 Institute of Pathology library creation pipeline.

Multimedia Appendix 2 Initial GEMINI query.

Multimedia Appendix 3 Revised GEMINI query.

Multimedia Appendix 4 Example of multipatient GEMINI query.

Multimedia Appendix 5 Example of combiner webservice JSON query.

Multimedia Appendix 6 Example of query request: SQL extension.

## Abbreviations

CDM: common data model
CSV: comma-separated values
DIC: data integration center
FHIR: Fast Healthcare Interoperability Resources
GEMINI: GEnome MINIng
GUI: graphical user interface
i2b2: informatics for integrating biology and the bedside
MI-I: Medical Informatics Initiative
MIRACUM: Medical Informatics in Research and Care in University Medicine
NGS: next-generation sequencing
OHDSI: Observational Health Data Sciences and Informatics
OMOP: Observational Medical Outcomes Partnership
SNP: single nucleotide polymorphism
SnpEff: single nucleotide polymorphism effect
SQL: structured query language
VCF: variant call format
