# Supplementary material for: Integrating Genomics and Clinical Data for Statistical Analysis by Using GEnome MINIng (GEMINI) and Fast Healthcare Interoperability Resources (FHIR): System Design and Implementation
Source: J Med Internet Res. 2020 Oct 7;22(10):e19879. doi: 10.2196/19879 (PMC7578821; doi:10.2196/19879)
Supplement: Multimedia Appendix 1 [file jmir_v22i10e19879_app1.pdf]

Multimedia Appendix 1 – Institute of pathology library creation pipeline

The Institute of Pathology of the University Hospital Erlangen uses a targeted NGS panel of 170 genes to identify non-synonymous gene variants in tumor specimens. In brief, tumor cells were micro dissected from freshly cut slides of formalin-fixed and paraffin-embedded tissue blocks to enrich for a tumor cell content of approximately 60%. DNA and mRNA were isolated separately, using the Maxwell 16LEV Blood DNA kit (Promega, Madison, USA) and the High Pure FFPET RNA Isolation Kit (Roche, Basel, Switzerland), respectively. For NGS-based mutation analysis, 40ng of extracted DNA and RNA were submitted to the enrichment-based TruSight Tumor 170 (TST170) library preparation kit (Illumina, San Diego, USA) according to the manufacturer's protocol. Once created, Libraries are sequenced on a Next Seq550 (Illumina) and analyzed for single nucleotide mutations, insertions, deletions, copy number variations, splice variants and gene fusions by using the TruSight Tumor 170 software (Illumina). Gene variants were annotated using Illumina`s VariantStudio v3.0. Identified gene variants are evaluated on their anticipated effects on tumor pathology using web tools and variant repositories, such as ClinVar, VarSome, JaxCKB, cBioPortal
