# Supplementary material for: Integrating Genomics and Clinical Data for Statistical Analysis by Using GEnome MINIng (GEMINI) and Fast Healthcare Interoperability Resources (FHIR): System Design and Implementation
Source: J Med Internet Res. 2020 Oct 7;22(10):e19879. doi: 10.2196/19879 (PMC7578821; doi:10.2196/19879)
Supplement: Multimedia Appendix 2 [file jmir_v22i10e19879_app2.pdf]

Multimedia Appendix 2 – Initial GEMINI query

```sql
SELECT
    *
FROM
    variants
WHERE
    filter IS NULL
    AND (
        impact_severity = 'HIGH'
        OR impact_severity = 'MED'
        OR (
            impact_severity = 'LOW'
            AND impact NOT IN (
                'stop_retained_variant',
                'synonymous_variant',
                '5_prime_UTR_variant',
                '3_prime_UTR_variant',
                'intron_variant',
                'upstream_gene_variant',
                'downstream_gene_variant',
                'intergenic_variant',
                'start_retained_variant',
                'conserved_intron_variant',
                'nc_transcript_variant',
                'non_coding_exon_variant'
            )
        )
    )
    AND aaf_1kg_all <= 0.02;
```
