# Supplementary material for: Integrating Genomics and Clinical Data for Statistical Analysis by Using GEnome MINIng (GEMINI) and Fast Healthcare Interoperability Resources (FHIR): System Design and Implementation
Source: J Med Internet Res. 2020 Oct 7;22(10):e19879. doi: 10.2196/19879 (PMC7578821; doi:10.2196/19879)
Supplement: Multimedia Appendix 3 [file jmir_v22i10e19879_app3.pdf]

Multimedia Appendix 3 – Revised GEMINI query

```
SELECT gene,codon_change, aa_change, gt_alt_freqs, clinvar_sig, clinvar_origin, aa_length, impact
FROM
   variants
WHERE
   filter IS NULL
   AND (
      impact_severity = 'HIGH'
      OR impact IN (
         'disruptive_inframe_deletion',
         'disruptive_inframe_insertion',
         'missense_variant'
      )
   )
   AND (
      clinvar_sig NOT IN (
         'benign',
         'likely_benign',
         'benign/likely_benign'
      )
      OR clinvar_sig IS NULL
   )
   AND aaf_1kg_all < 0.02;
```
