# Supplementary material for: Integrating Genomics and Clinical Data for Statistical Analysis by Using GEnome MINIng (GEMINI) and Fast Healthcare Interoperability Resources (FHIR): System Design and Implementation
Source: J Med Internet Res. 2020 Oct 7;22(10):e19879. doi: 10.2196/19879 (PMC7578821; doi:10.2196/19879)
Supplement: Multimedia Appendix 4 [file jmir_v22i10e19879_app4.pdf]

## Multimedia Appendix 4 – Example Multi Patient GEMINI query

```
SELECT gene, impact, codon_change, SUM(num_het + num_hom_alt)
FROM variants v1
WHERE gene IS NOT NULL
     AND (impact_severity = 'MED' OR impact_severity = 'HIGH')
GROUP BY gene, impact, codon_change
HAVING sum_mutations_for_all_patients > 1
      AND SUM(num_het + num_hom_alt) IN
      (SELECT SUM(num_het + num_hom_alt)
       FROM variants
       WHERE gene = v1.gene
            AND (impact_severity = 'MED' OR impact_severity = 'HIGH')
       GROUP BY gene, impact, codon_change
       ORDER BY sum_mutations_for_all_patients DESC
       LIMIT 3 )
ORDER BY gene,sum_mutations_for_all_patients DESC;
```
