# Supplementary material for: Integrating Genomics and Clinical Data for Statistical Analysis by Using GEnome MINIng (GEMINI) and Fast Healthcare Interoperability Resources (FHIR): System Design and Implementation
Source: J Med Internet Res. 2020 Oct 7;22(10):e19879. doi: 10.2196/19879 (PMC7578821; doi:10.2196/19879)
Supplement: Multimedia Appendix 5 [file jmir_v22i10e19879_app5.pdf]

Multimedia Appendix 5 – Example combiner web service JSON query


```json
{
    "format": "csv",
    "patient_ids": [
        1,
        2,
        ...,
        100
    ],
    "fhir": {
        "patient": [
            {
                "resource_val_path": "gender"
            },
            {
                "resource_val_path": "birthDate"
            }
        ],
        "observation": [
            {
                "key": "code.coding.code",
                "value": "28293008"
            }
        ]
    },
    "gemini": {
        "database": {
            "merged": true
        },
        "variants": {
            "columns": [
                "ref",
                "alt",
                "gts"
            ],
            "keys": [
                {
                    "chrom": "chrX",
                    "start": "154158284",
                    "end": "154158285",
                    "alt": "C"
                },
                ...
            ]
        }
    }
}
```
