# Supplementary material for: Integrating Genomics and Clinical Data for Statistical Analysis by Using GEnome MINIng (GEMINI) and Fast Healthcare Interoperability Resources (FHIR): System Design and Implementation
Source: J Med Internet Res. 2020 Oct 7;22(10):e19879. doi: 10.2196/19879 (PMC7578821; doi:10.2196/19879)
Supplement: Multimedia Appendix 6 [file jmir_v22i10e19879_app6.pdf]

Multimedia Appendix 6 – Example query request – sql extension


```
"GEMINI": {
    "database": {
        "merged": false
    }
"sql_mode": true,
    "database": {
        "merged": false
    },
    "sql": "select distinct gene from variants where filter IS NULL AND(impact_severity='HIGH' OR impact
IN('disruptive_inframe_deletion','disruptive_inframe_insertion','missense_variant')) AND (clinvar_sig NOT IN
('benign','likely_benign','benign/likely_benign') OR clinvar_sig IS NULL) AND aaf_1kg_all<0.02",
    "columns": [
        "gene"
    ],
    "rows": [
        "gene"
    ]
}
```
